# Bacteriocins from Lactic Acid Bacteria. A Powerful Alternative as Antimicrobials, Probiotics, and Immunomodulators in Veterinary Medicine

**DOI:** 10.3390/ani11040979

**Published:** 2021-04-01

**Authors:** Juan Carlos Hernández-González, Abigail Martínez-Tapia, Gebim Lazcano-Hernández, Blanca Estela García-Pérez, Nayeli Shantal Castrejón-Jiménez

**Affiliations:** 1 Área Académica de Medicina Veterinaria y Zootecnia, Instituto de Ciencias Agropecuarias, Universidad Autónoma del Estado de Hidalgo, Av. Universidad km 1 Exhacienda de Aquetzalpa A.P. 32, Tulancingo 43600, Mexico; juan_hernandez8281@uaeh.edu.mx (J.C.H.-G.); ma335862@uaeh.edu.mx (A.M.-T.); la319548@uaeh.edu.mx (G.L.-H.); 2 Department of Microbiology, Escuela Nacional de Ciencias Biológicas, Instituto Politécnico Nacional, Prolongación de Carpio y Plan de Ayala S/N, Col. Santo Tomás, México City 11340, Mexico; blgarciap@ipn.mx

**Keywords:** bacteriocins, antimicrobials, lactic acid bacteria, probiotics, immunomodulation, veterinary medicine

## Abstract

**Simple Summary:**

Antibiotic resistance is a growing threat; its indiscriminate use has led to management restrictions in humans and animals. Bacteriocins are powerful antimicrobial peptides that have great potential in the prevention and treatment of diseases in animals. Their antimicrobial activity is rapid, and they show a lower propensity to develop resistance than conventional antibiotics. Currently, their main application is in food preservation systems. However, several studies show their bioactive role as antimicrobials, probiotics, and immunomodulators in animals. Therefore, bacteriocins are an excellent alternative to be applied in several areas of veterinary medicine.

**Abstract:**

In the search for an alternative treatment to reduce antimicrobial resistance, bacteriocins shine a light on reducing this problem in public and animal health. Bacteriocins are peptides synthesized by bacteria that can inhibit the growth of other bacteria and fungi, parasites, and viruses. Lactic acid bacteria (LAB) are a group of bacteria that produce bacteriocins; their mechanism of action can replace antibiotics and prevent bacterial resistance. In veterinary medicine, LAB and bacteriocins have been used as antimicrobials and probiotics. However, another critical role of bacteriocins is their immunomodulatory effect. This review shows the advances in applying bacteriocins in animal production and veterinary medicine, highlighting their biological roles.

## 1. Introduction

Bacteriocins are heat-stable, ribosomally synthesized antimicrobial peptides. Both Gram-positive and Gram-negative bacteria, and archaea release antimicrobial peptides extracellularly in the late-exponential to the early-stationary growth phases [1]. An essential attribute of bacteriocins is the antimicrobial activity against different bacteria, fungi, parasites, viruses, and even against natural resistant structures, such as bacterial biofilms [2,3,4,5]. Lactic acid bacteria (LAB) are a heterogeneous group of Gram-positive bacteria. They are classified according to glucose fermentation characteristics, cell morphology, capacity to utilize sugars, and optimum growth temperature range [6]. Thus, this classification system recognized a core group consisting of four genera: *Lactobacillus*, *Pediococcus*, *Leucononstoc*, and *Streptococcus* [7]. Molecular biological methods have increased the number of genera, including the following: *Aerococcus*, *Alloiococcus*, *Carnobacterium*, *Dolosigranulum*, *Enterococcus*, *Lactococcus*, *Lactosphaera*, *Melissococus*, *Oenococcus*, *Tetragenococcus*, *Vagococcus*, and *Weissella* [6,8]. Various studies have shown that LAB inhibit pathogenic microorganisms growth, degrade mycotoxins, and have a probiotic effect [6]. LAB are found abundantly in nature and symbiotically interact with higher organisms. They have been isolated from several sources, including dairy products, meat, fruits, and vegetables. They can also be found in mucous membranes of the respiratory, intestinal, and other anatomical sites of man and animals, even in plants, wastewater, soil, and manure [9]. Bacteriocins have been used as food preservatives, due to their ability to inhibit microorganisms potentially harmful to human health. They are safe for consumption and do not alter the quality and safety of food [10,11]. Furthermore, bacteriocins from LAB have had a significant development in other fields, such as in the cosmetic industry and human and veterinary medicine [12,13]. In animal production, bacteriocin-producing bacteria have been used as probiotics in the diet or drinking water of pigs, poultry, and fish, which has increased their growth rate [14,15].

LAB are among the important groups of bacteria that provide health benefits for humans and animals. Therefore, in this review, we focus on showing the advances in applying LAB bacteriocins as antimicrobials, probiotics, and immunomodulators in animal production and veterinary medicine.

## 2. Bacteriocin Overview

Bacteriocins are a diverse group of antimicrobial cationic and hydrophobic peptides composed of 20–60 amino acids. The ribosomal machinery is responsible for the synthesis of bacteriocins. Several genes are implicated in modifying amino acids, the export and regulation of the bacteriocin, and self-immunity proteins [16,17,18]. Bacteriocin encoding genes are organized into operons located in the chromosome, plasmids, or other mobile genetic elements. In general, these operons are inducible and require secretion and extracellular accumulation of bacterial peptides for induction [19,20]. Bacteriocins are extracellularly released and can have bactericidal or bacteriostatic effects on species closely related to the producing strain or affect other genera, phylum, or even domain [21,22].

Furthermore, the influence of environmental factors promotes the secretion of bacteriocins, including bacterial cell density, nutrient availability, the presence of acetic acid, and signaling peptides (competence stimulating peptide molecules) [23]. Interestingly, bacteriocins have been reported to be 10^3^–10^6^ times more potent than various other antimicrobials, including conventional antibiotics [13]. Therefore, bacteriocins-producing bacteria synthesize self-immunity proteins that protect them from their bacteriocins by scavenging bacteriocins or antagonist competition for receptor bacteriocin [17,24,25]. An important advantage of bacteriocins is that they can have activity against pathogenic and opportunistic bacteria, including multidrug-resistant species, without discriminating between antibiotic-resistant and sensitive strains [26]. Several bacteriocins have been shown to act in synergy with conventional antibiotics, reducing concentrations, undesirable side-effects, and the prevalence of resistant strains [27].

Interestingly, the combination of bacteriocins and antibiotics has been proposed as novel therapeutic options for food-producing animals. The possibility of replacing the use of antibiotics is explored to avoid bacterial resistance. Various reports have also established LAB bacteriocins advantages and synergistic actions with other biomolecules, such as nisin and citric acid, against *Staphylococcus aureus* and *Listeria monocytogenes* [28]. It has also been documented that bacteria can develop resistance to bacteriocins. However, resistance to bacteriocins is minimal compared to conventional antibiotics. Since the frequency of spontaneous mutations in cells exposed to bacteriocins is low [28]. This resistance is generally through modifications in the cell envelope, such as alterations in the charge and thickness [20,29,30].

## 3. Classification of Bacteriocins

Numerous bacteriocins have been isolated from LAB and are described in several databases. They have different characteristics, structures, modes of action, biochemical properties, activity spectra, and target cell receptors [31,32]. Bacteriocins produced by Gram-positive bacteria have been classified into three groups according to their biochemical and genetic characteristics or the presence of disulfide or monosulfide bonds, molecular weight, thermal stability, proteolytic enzymatic stability, presence or absence of post-translational modification of amino acids, and antimicrobial action [33] (Table 1). Initially, a fourth class of bacteriocins was described; however, it has been aborted and renamed as bacteriolysins, which comprise large complexes with carbohydrate and lipid residues [34,35].

Class I bacteriocins (lantibiotics) are small peptides (<5 kDa), 19 to 50 amino acids, are heat-stable, and have a post-translational modification, resulting in the formation of atypical amino acids lanthionine and methyllanthionine. These modify and introduce intramolecular cyclic structures, providing rigidity and resistance to the action of proteases [36,37,38]. Further, class I is divided into three subclasses: Class Ia comprises flexible, elongated, positively charged, and hydrophobic peptides associated with a pore formation in bacterial membranes; the most representative bacteriocin of this group is nisin [33]. Class Ib is made up of globular and inflexible bacteriocins that are negatively charged or have no net charge. These peptides can inhibit catalytic enzymes essential for the survival of susceptible bacteria [39]. Class Ic (sactipeptides) are sulfur-to-α-carbon-containing peptides. No bacteriocin of this group from LAB has been characterized [38].

Class II (nonlantibiotic) bacteriocins are small and flexible (<10 kDa), with an amphiphilic helical structure. These peptides do not contain modified amino acid residues and are pH and heat-resistant. Class II bacteriocins are divided into four subclasses based on structure and modifications. Class IIa bacteriocins (pediocin-like) include peptides from 35 to 50 amino acids that contain the YGNGV consensus sequence at the N-terminus. These bacteriocins have potent activity against *L. monocytogenes* [13,40,41]. Class IIb (two-peptide unmodified bacteriocins) consists of two different complementary peptides. The optimal antimicrobial activity requires both peptides that are members of this group in about equal amounts [42]. Class IIc (circular bacteriocins) contains 35 to 70 amino acids. These bacteriocins are associated with a leader peptide sequence and include one to two cysteine residues in their structure; they are further divided into cystibiotics and thiolbiotics. These peptides are resistant to many proteolytic enzymes [43,44,45]. Class IId comprises linear, non-pediocin-like, single-peptide bacteriocins [46].

Class III bacteriocins have high molecular weight (>30 kDa) and are thermolabile and unmodified peptides. They have a bacteriolytic (class IIIa) or nonlytic mechanism of action (class IIIb). These bacteriocins have been poorly studied [47,48].

## 4. Mechanism of Action of Bacteriocins

**Figure 1:**
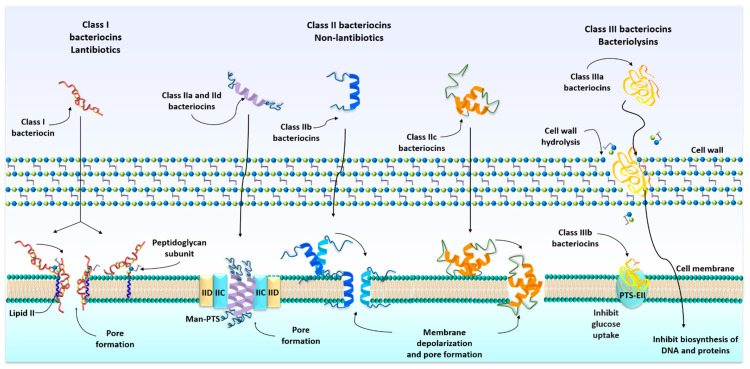
Mode action of bacteriocins. Bacteriocins act directly on the membrane or through a specific receptor on the target cell and form pores in the bacterial cell membrane, which leads to cell death.

The mechanism of action of bacteriocins depends on their primary structure. Some can exert their activity on the cytoplasmic membrane releasing compounds vital of susceptible bacterial (cell lysis); others can enter the cytoplasm and affect gene expression and protein synthesis (Figure 1) [26].

Lantibiotics (class I) bacteriocins have a dual mode of action. They inhibit bacterial cell wall biosynthesis through binding to lipid II, a hydrophobic carrier of peptidoglycan monomers, from the cytoplasm to the cell wall, compromising cell viability. Additionally, lantibiotics can use lipid II as a docking molecule to initiate a process of membrane insertion and pore formation in the bacterial membrane [49,50,51].

Non-lantibiotics (class II), such as pediocin-like and the one-peptide nonpediocin-like bacteriocins (class IIa and class IId), bind to MptC and MptD subunits of the mannose phosphotransferase permease (Man-PTS). The insertion of these bacteriocins into the target cell membrane leads to an irreversible opening of an intrinsic channel, which leads to the diffusion of ions through the membrane, causing the death of the target cell [41,52,53]. Class IIb, two-peptide unmodified bacteriocins, permeabilize the membrane of sensitive bacteria and form pores. These pores show specificity for monovalent cations, such as Na^+^, K^+^, Li^+^, Cs^+^, and Rb^+^ (described in lactococin G) [41,54]. Circular bacteriocins (class IIc) have a positive net charge. These peptides interact directly with the negatively charged bacterial membrane without requiring any receptor molecules. Consequently, pores are formed in the cell membrane, causing ions efflux and the dissipation of the membrane potential, leading to cell death [55,56]. Bacteriolysins (class IIIa bacteriocins) catalyze cell wall hydrolysis, resulting in cell lysis [48,57]. Nonbacteriolytic bacteriocins (class IIIb) exert their action by disturbing the glucose uptake by cells, starving them, and disturbing the membrane potential. Another mechanism is inhibiting the biosynthesis of DNA and proteins of target bacteria [23,58,59].

## 5. The Antimicrobial Properties of Bacteriocins in Veterinary Medicine

LAB bacteriocins have great potential in veterinary medicine. Several in vitro and in vivo studies have evaluated their therapeutic use in small animals, livestock, and poultry (Table 2). This evidence demonstrates that bacteriocins can be applied as a substitute for antibiotics and represent an alternative as new antimicrobials that prevent bacterial resistance [22,60]. 

Nisin is an antimicrobial cationic peptide produced by *Lactococcus lactis* subsp. *lactis* and *Streptococcus* species. Nisin was the first antimicrobial agent in reaching the category of food-safe additive in 1969. The use of nisin is, at present, licensed in more than 50 countries [61]. The United States granted the Generally Regarded as Safe (GRAS) designation by the FDA [50]. Researchers have shown that the antimicrobial action of nisin can extend to non-food-related bacteria. Thus, nisin has been included in clinical studies to prevent the formation of dentobacterial plaque and gingivitis in dogs, with an effect comparable to chlorhexidine [62,63]. An advantage of its use is the stability to remain active and express a synergistic action among a series of topical delivery systems: mouthwash, toothpaste, and guar-gum biogel [62,63]. In dogs, diverse oral bacteria species form a polymicrobial biofilm in the tooth surface, developing periodontal disease (PD) [64]. *Enterococcus faecalis* is the main bacteria in the oral cavity of dogs involved in PD, and it shows resistance to different antibiotics [65]. Further, *E. faecalis* leads to adherence and aggregation of other pathogen bacteria, all of them forming the biofilm of PD [66]. Pet animals have been considered reservoirs of this species of potentially pathogenic bacteria that is potentially harmful to high-risk individuals [67,68,69]. Nisin shows broad bactericidal action in vitro against reference strains of *E. faecalis* and other bacteria involved in canine PD. The combination of nisin–biogel inhibits and eradicates canine PD multispecies biofilms tested in a model of co-aggregate bacteria in vitro with *E. faecalis*, *Neisseria* *zoodegmatis*, *Corynebacterium canis*, *Porphyromonas cangingivalis*, and *Peptostreptococcus canis* [66]. Prolonged nisin use in oral canine cleaning does not have the negative effects that chlorhexidine exhibits, such as taste loss or pigmentation of the enamel [70,71,72]. *Enterococcus faecium* is another bacterium implicated in PD disease; it is considered a zoonotic opportunist, and it is also the most abundantly isolated from the feces of healthy dogs [73]. Paradoxically, some isolates of *E. faecium* produce bacteriocins like enterocin A, B, and P, which inhibit the growth of *L. monocytogenes* [74,75]. 

New alternatives in livestock are being sought to replace antibiotics and diminish bacteria resistant to them. Nisin has been used as a treatment in bovine mastitis caused by complex bacterial: *Enterococcus* spp., *Staphylococcus* spp., and *Streptococcus* spp. [76,77]. The main advantage is that nisin only remained in the milk for 12 h after its application in concentrations that did not generate any risk in consuming the product, aside from avoiding bacterial resistance [78,79]. Due to this, the FDA approved a nisin-based udder disinfectant [33]. In addition, nanoparticles to which nisin was included have shown a bactericidal effect against multidrug-resistant *Staphylococcus* spp. isolated from bovine mastitis and methicillin-resistant *S. aureus* [80,81]. Some species of *S. aureus* produce biofilm, a virulence factor that favors the establishment of infection in the udder and interferes with antibiotics [82]. A study demonstrated the antimicrobial activity of nisin on biofilm-producing *S. aureus* cultures. Furthermore, it penetrates the biofilm matrix, which leads to the detachment of the biofilm. Likewise, the combination of nisin and lysostaphin resulted in synergy against *Staphylococcus* spp. biofilm [83]. Nisin alters the biofilm’s architecture and composition, reducing the polysaccharides that constitute the biofilm of *S. aureus* and *Staphylococcus epidermidis* without affecting the integrity of the proteins. In addition, nisin reduced the extracellular DNA in *S. aureus* biofilm, but this effect was not observed in the *S. epidermidis* biofilm [84]. In pig production, there are opportunistic pathogens, such as *Streptococcus* and *Escherichia coli* species. *Streptococcus suis* serotype 2 is a zoonotic pathogenic that causes great economic losses in the pig industry. The co-administration of nisin with conventional antibiotics for treating swine *Streptococcus* infection can decrease antibiotic resistance. This bacteriocin elicits the bacterial membrane permeability to facilitate the antibiotic to reach the target [85]. In addition, nisin can cross the capsule of *S. suis*, degrade biofilm, and kill the bacteria. In mice infected by *S. suis*, nisin reduced bacterial load and avoided bacterial systemic dissemination [86]. Nisin V, a variant of nisin (bioengineering synthesized), showed enhanced potency against *L. monocytogenes* in vivo. Nisin V decreased the load of *Listeria monocytogenes* in the liver and spleen of mice experimentally infected [87]. Avirulent strains of *S. suis* serotype 2 (isolated from the tonsils of healthy pigs) produce bacteriocins suicin 90–1330, suicin 65, and suicin 3908. These antimicrobial peptides have an antibacterial effect on virulent *S. suis*, including bacteria resistant to erythromycin and tetracycline. Interestingly, structure amino acid sequences within these suicins have little similarity to each other (<25%) [88,89,90]. A recombinant suicin has shown inhibitory activity against Gram-positive strains [91]. Enterotoxigenic *E. coli* causes diarrhea in piglets. The use of colistin (polymyxin E) has led to the development of a resistant strain [92,93,94]. The combination of nisin or enterocin DD14 with colistin showed a synergistic effect against colistin-resistant *E. coli* strains isolated from pigs. This effect is because the loss of membrane stability induced by interacting colistin with LPS allows the entry of bacteriocins to damage the cell wall [95]. In horses, enterocin M in the diet reduced undesirable Gram-negative bacteria: coliforms, *Campylobacter*, and *Clostridium* spp. No physiological parameter was altered by the administration of enterocin M in horses [96]. Nisin added to water in weaned rabbits decreases harmful intestinal microbiota: *Staphylococcus* coagulase-positive, coliforms, *Pseudomonas*, and Clostridiae. Rabbits with nisin treatment increased their body weight average, and meat quality was not affected [97,98]. A partially purified bacteriocin PPB CCM7420, isolated from *E*. *faecium*, showed a significant reduction of parasite *Eimeria* spp. oocysts in rabbits [98].

Some types of nisin are effective against aquaculture pathogens. Araújo et al. (2015) demonstrated that the Nisin Z (produced by *L. lactis* subsp. *cremoris*) prevents lactococcosis in rainbow trout. *Pediococcus acidilactici* L-14 produces Pediocin PA-1 bacteriocin, which was shown to have antimicrobial activity against fish pathogens, such as *Lactococcus garvieae*, *Streptococcus iniae*, *Carnobacterium maltaromaticum*, and *Aeromonas salmonicida* [99]. Treatment with enterocin AS-48 in trout infected by *L. garvieae*, led to an outcome of a survival rate of 60%, compared to untreated fish that did not survive [100]. Other studies suggest that *Lactobacillus pentosus* HC-2 and *E. faecium* NRW-2 could be used in the shrimp diet, as they have antibacterial activity against *Vibrio harveyi* and *Vibrio parahaemolyticus* (ATCC 17802) [101]. The combination of bacteriocins from LAB and eukaryotic antimicrobial peptides (AMP) showed a synergistic activity and broadened the spectrum range. It has been shown that pediocin PA1, sakacin P, and curvasin A increased the bactericidal activity of pleurocidin and AMP of fish against *E. coli*. These bacteriocins also have high antimicrobial activity against *Listeria ivanovii* [102]. The use of nisin and OaBac5mini (ovine cathelicidin) increased the bactericidal activity against methicillin-resistant *S. aureus* 1056 [103].

The lantibiotic lacticin produced by *L. lactis* subsp. *lactis* DPC3147 (GRAS) is active against potential pathogens of veterinary importance, including methicillin-resistant *S. aureus*, *Streptococcus dysgalactiae*, *Streptococcus uberis*, vancomycin-resistant *E. faecalis*, *Clostridium difficile*, *Mycobacterium avium* subsp. *paratuberculosis*, *L. monocytogenes*, and others [104,105,106,107,108,109,110]. Some of the most promising research for the use of this lantibiotic is for the treatment of mastitis. Currently, the treatment of choice for bovine mastitis involves the use of commercial therapeutic antibiotic formulations. However, a recent study shows that *L. lactis* DPC3147 (which produces lacticin 3147) used to treat cows with clinical/subclinical mastitis showed efficacy comparable to that of antibiotic treatment (kanamycin and cephalexin) [109]. Due to the excellent antimicrobial activity exhibited by lacticin 3147, the application of a teat seal that contains a combination of bacteriocin and bismuth to prevent *S. dysgalactiae* infection in dry cows and *S. aureus* infection in lactating cows has been proposed [110,111]. The lacticin NK34 is a variant that adds to mastitis control by bacteriocins. It has shown in vitro high antimicrobial activity against *S. aureus* and coagulase-negative *Staphylococcus* strains isolated from bovine mastitis. In an experimental infection in mice with *S. aureus*, antimicrobial activity of lacticin NK34 ensures survival above 80% [112]. 

The antimicrobial property of bacteriocins has been exploited to control the pathogenic microbiota in poultry. Plantaricin (isolated from *Lactobacillus plantarum* F1) was proposed as a viable alternative to replacing the use of antibiotics against colibacillosis in broiler chickens [113]. Plantaricin CLP29 and enterocin CLE34, partially purified, have wide antibacterial activity against *Salmonella pullorum* and *E. coli* [114]. The discovery of new bacteriocins for application in poultry has been made in the microbiota of the domestic broiler chicken’s gastrointestinal tract. The bacterium *Paenibacillus polymyxa* NRRL B-30509 was isolated from domestic Russian broiler chickens, producing the bacteriocin paenicidin A, which has activity against *Campylobacter jejuni* [115]. Another bacteriocin with potential use in poultry is pediocin A (isolated from *Pediococcus pentaceus* FBB61) [116]. The antimicrobial activity has been reported against Gram-positive bacteria, such as *L. monocytogenes* and *Clostridium perfringens* type A [117]. In broilers infected by *C. perfringens* type A (producer of NetB toxin) involved in necrotic enteritis, Pediocin A was administered in food. The treatment improved the growth performance of the chickens. However, it did not decrease the bacterial load [118]. Microencapsulation of bacteriocin could prevent the inactivation of bacteriocin, due to digestive processes in broilers [119]. The combination of bacteriocins divercin AS7 and nisin as an additive in the diet of broilers has been shown to improve body weight gain [120,121]. Divercin AS7 and nisin showed bactericidal activity similar to salinomycin (ionophore coccidiostat) [121]. Nisin has antimicrobial action on microbiota related to the detriment of productivity in broiler chickens, similar to monensin ionophore. Nisin supplementation positively affected the gut microbiota by reducing potentially pathogenic bacterial populations in the jejunum and ceca, such as Enterobacteriaceae, *C. perfringens*, *Clostridium coccoides*–*Eubacterium rectale* cluster, *Bacteroides*–*Prevotella* cluster, *Lactobacillus* sp./*Enterococcus* sp., and the *Clostridium leptum* subgroup [122]. The diminished load of bacteria associated with low productivity in the gastrointestinal tract decreases the competition of nutrients and improves energy use in chickens [123]. These findings highlight the role of bacteriocins as an excellent antibacterial alternative against potentially pathogenic agents for animals and improved growth performance.

## 6. The Probiotic Activity of LAB Bacteriocins

Oral administration of purified or semipurified bacteriocins has been shown to have limitations. Digestive enzymes can degrade bacteriocins, and bacteriocins can adhere to food particles or diffuse through digestion, among others [126]. The protection of bacteriocins in capsules or nanocapsules can be an alternative to prevent enzymatic degradation and avoid various doses and high concentrations of bacteriocins [127]. Thus, the most efficient method for taking advantage of bacteriocins in the digestive tract includes bacteriocin-producing LAB as probiotics. This strategy favors the colonization of bacteria in the gastrointestinal tract, and bacteriocins can be produced in situ [128]. There are multiple benefits of using LAB in place of antibiotics or growth promoters. These include modulation of microbiota, improving the intestinal barrier function and digestion, preventing the colonization of enteric pathogens, and stimulating the immune system [15,129,130,131]. However, studies showed that *E. faecium* LMG 30881, a producer of enterocin B in the canine diet, caused unfavorable effects, such as runny stools, higher Gram-negative bacterial counts, and lower hemoglobin concentrations [132,133]. The use of bacteriocin-producing LAB probiotic in dogs requires more research, since no further work has been generated in this regard to date. In healthy pigs, it has been shown that some LAB of the gastrointestinal tract prevents villous atrophy of the post-weaning stage, promotes the maturation of gastrointestinal lymphoid tissue, and has immunomodulatory activity. Oral administration of the probiotic *Lactobacillus salivarius* B1 (isolated from healthy piglets) in newborn piglets showed that the probiotic bacteria colonized the duodenal mucosa and increased the height of the villi, which improved absorption and promoted the integrity of the intestinal barrier. Interestingly, *L. salivarius* increased the expression of porcine beta-defensin 2 (pBD-2), an antimicrobial peptide produced by host cells. Continuous probiotic administration caused a considerable increase in the production of pBD-2, which could even be detected in the saliva of piglets [134]. In the duodenum and ileum, the number of intraepithelial lymphocytes, plasma cells that produce IgA, and the synthesis of Toll-Like Receptor-2 (TLR-2) increased. In the ileum, interleukin-6 (IL-6), a cytokine that promotes the differentiation and proliferation of B lymphocytes, was increased [130]. These studies suggest that the immunomodulatory effects of *L. salivarius* B1 are due to bacteriocins [135]. *L. salivarius* UCC118 (isolated from the human intestinal microbiota) produces the bacteriocin Abp118. This antimicrobial peptide has been shown to have activity against *L. monocytogenes* [136,137]. *L. salivarius* UCC 118, as a probiotic added to the diet of pigs after weaning, showed that LAB colonized the ileum and caused a significant decrease in spirochetes (*Treponema*), considered to be opportunistic pathogens of pigs [138]. Rustic or native animals on farms can be a natural source of bacteriocins-producing probiotics. A study has shown that miniature piglets from Congjiang (a breed of pig native to China) had higher resistance to stress-induced diarrhea during early weaning due to gut microbiota. In the feces of miniature piglets, a higher population of *Lactobacillus gasseri* LA39 and *Lactobacillus frumenti*, producers of the bacteriocin gassericin, was found. This study also demonstrated an increase in the signaling pathway involved in protein expression (NHE3, SLC5A1, DRA, and PAT1) associated with intestinal absorption. Gassericin decreased the expression of proteins related to intestinal secretion (NKCC1, CFTR, CaCC1). These bacteria could be transplanted into commercial crossbred piglets before weaning and prevent diarrhea after weaning. This study provides a strategy for the possible prevention of diarrhea in pigs and even other mammals [139]. There are reports of more than 30 LAB as probiotics that inhibit the growth of pathogenic microorganisms in birds; however, their mode of action remain poorly understood [140,141,142]. LAB as probiotics in poultry has been used to control experimental coccidial infection, endemic in the commercial broiler industry. Studies on the inclusion of multispecies probiotics (*E*. *faecium*, *Bifidobacterium animalis*, and *L*. *salivarius*) in food or water have shown that the colonization of LAB in the intestine causes a coccidiostatic effect on *Eimeria* spp. In addition, the probiotic prevented intestinal damage without affecting body weight gain values. On the other hand, there were high probiotic LAB amounts in intestinal microbiota, while coliform and *C. perfringens* were lower than the control group [143]. The multispecies probiotic for commercial use can be integrated into chicken microbiota early in ovo and for one day of hatching. These bacteria increased protection against *Eimeria* spp. and commercial vaccine-administered response, at the same time [144,145]. The species of genera *Enterococcus* spp., as a probiotic for use in the poultry industry, has been shown to produce enterocin A, B, P, and L50 and bacteriocin-like inhibitory substances that are not identified currently. These bacteriocins have demonstrated antimicrobial activity in vitro against pathogenic bacterial *C. perfringens*, *S. aureus*, *Salmonella* Heidelberg, and *L. monocytogenes* [146,147].

## 7. Bacteriocins as Immunomodulators

The immunomodulatory effect of bacteriocins has not been fully elucidated. It is known that modulation of the immune system depends on the concentration of bacteriocin used. These activation mechanisms of the immune response by bacteriocins add to the bactericidal effect, thereby increasing host protection, particularly during infections. The lantibiotic nisin is the oldest and most widely used bacteriocin in the food industry [148]. Nisin has shown that its administration in the diet for short periods increases CD4+ and CD8+ T lymphocytes (LT) and reduces the lymphocytes B (LB) levels in the blood. Its consumption in prolonged period results in return to normal LT levels, the maintained decrease in LB, and the count increase of macrophages/monocytes [149]. The high concentration of nisin added to porcine PBMC (Peripheral Blood Mononuclear Cells) in vitro equally increased CD4+ and CD8+ proliferation and cytokine IL-1β and IL-6 production [150]. In rabbit’s vaginal tissue explant culture, nisin showed high biological compatibility with tissue and did not show any immunomodulatory effect. The lacticin did not affect the expression of defensin, TLR3, or TLR9 receptors, nor the expression of cytokines IL-4, IL-6, GM-CSF, IL-8, IL-6, or TNF-α [151,152]. Moreover, in neutrophils, high concentrations of nisin activate extracellular traps (NETs) and increase intracellular superoxide levels [153]. Interestingly, although the antimicrobial activity of nisin in vitro is limited to Gram-positive bacteria, when administered to animals infected by Gram-positive and Gram-negative bacteria, the host’s bacterial load significantly decreases. In these cases, experiments with human PBMC explain that the protection of nisin is due to chemokines (MCP-1, IL-8, and Gro-α) that represses proinflammatory TNF-α production. In this experiment, nisin shows greater potency than human antimicrobial peptide LL-37 [154]. Nisin modulates some nonspecific immune functions in turbot. The treatment with intermediate and lower doses of nisin in turbot head kidney macrophages increased oxygen free radical production and phagocytic function and did not affect nitric oxide production. The lowest doses of nisin injection in turbot augmented lysozyme concentration in serum [155].

The immunomodulatory effect of nisin is expressed even in nonimmune cells. In bovine mammary epithelial cells, nisin increases intracellular lysozyme and even releases it to the extracellular environment [156]. Bacteriocins as immune-modulating agents have shown anti-inflammatory properties in damaged or infected tissue. In LPS-stimulated PBMC, nisin inhibits the synthesis of TNF-α, thereby decreasing the inflammatory response [154]. In porcine PBMC infected by *E. coli*, nisin decreased the inflammatory response, mainly the production of IL-6 [150]. This anti-inflammatory effect is reproduced in bovine mammary gland epithelial cells by promoting a negative regulation in the production of TNF-α, which benefits the recovery of intramammary tissue. Endometritis represents a frequent health problem in the first three weeks postpartum. In bovines, nisin prevents endometritis induced by experimental infection with *S. aureus*. Bacteriocins in this infection promote a decrease in the proinflammatory cytokines and increase the anti-inflammatory cytokines [157]. In tissue injuries, such as fractures, the bacteriocins produced by *Lactobacillus rhamnosus* L34 and *L. rhamnosus* (ATCC 53103) were shown to reduce postoperative effects, such as inflammation. Additionally, they favor the control of experimental intra-articular infection with *S. aureus* [158,159]. Bacteriocins from *L. rhamnosus* caused a decrease in proinflammatory cytokines TNF-α, IL-6, and C-reactive protein in rabbit models of mandible fracture fixation and knee re-placement surgery. In addition, they controlled experimental *S. aureus* infection by minimizing biofilm formation and promoting tissue repair [158,159]. These results suggest that bacteriocins could be potent agents for preventing postoperative orthopedic infections. A recent study demonstrated that the LAB *Pediococcus pentosaceus* (SL001) express the bacteriocin coagulin. A supplemented diet with *P. pentosaceus* enhanced the immunity of grass carp, increasing IgM and C3 (complement 3), whereas IL-8 was downregulated. Moreover, *P. pentosaceus* contributed to the elimination of pathogens and promoted grass carp growth rate [160].

Interestingly, the bacteriocins produced by pathogens do not have a bactericidal effect. These bacteriocins seriously affect the host’s immune system, favoring disease. *Streptococcus iniae* is a pathogen that affects fish and humans and produces the bacteriocin Sil. Sil has only shown bacteriostatic activity against *Bacillus subtilis* and is not cytotoxic in healthy fish tissues. However, Sil bacteriocin administration to fish before *S. iniae* infection reduced respiratory burst and acid phosphatase activity in turbot head kidney monocytes. Furthermore, the bacterial infection spread to the kidney and spleen [161].

## 8. Conclusions

Bacteriocins are a powerful weapon that can be exploited in veterinary medicine. The administration of these antimicrobial peptides in domestic animals eliminates potentially pathogenic undesirable microorganisms without causing cytotoxicity on cells or tissues. Interestingly, it does not generate resistance to antibiotics, and resistance to bacteriocins is minimal. Bacteriocins are analogous and synergistic when combined with antiseptics, antibiotics, and ionophores, showing greater potency than antimicrobial peptides from eukaryotic cells. In addition, these combinations can reduce resistance to bacteriocins. The potential use of bacteriocins alone or along with microbicidal agents has potential therapeutic actions in periodontal disease and mastitis in dairy cows, prevents postoperative infections in fractures, and coccidiostats and improves productive parameters in substitution of antibiotics as growth promoters. In animal nutrition, bacteriocins reduce cholesterol and triglycerides, thus improving the quality of the meat as a final product. LAB colonize the intestinal mucosa and produce bacteriocins in situ. LAB and their bacteriocins promote the integrity of the intestinal barrier, eliminate bacteria that interfere with the use of nutrients, and stimulate the expression of proteins associated with the absorption of intestinal fluid. This finding suggests that they can be used as probiotics in poultry and monogastric animals. Furthermore, these bioactive peptides have a role in the immune response as immunomodulators. Bacteriocins modulate the expression of anti-inflammatory cytokines to promote the repair of cell damage. Some of them are potent inducers of antimicrobial peptides in eukaryotic cells, which improve the innate immune response against pathogens. However, the bacteriocins produced by virulent bacteria can be a virulence factor, promoting a proinflammatory cytokine profile, which favors infection in lymphoid cells and organs. Little is known about the immunomodulatory effects of bacteriocins in animals. More studies are required to fully understand the role of bacteriocins on the innate and adaptive response that could contribute to the control or resolution of infections or diseases. LAB bacteriocins are projected as new antimicrobials that could be targeted or stabilized by nanotechnology and prevent enzymatic digestion. In addition, more research is required on modifications that could increase the potency of bacteriocins. The benefits of bacteriocins shown in vitro and in vivo assays provide support for developing and researching clinical trials in the different areas of veterinary medical therapeutics.

## Figures and Tables

**Table 1:** Classification of bacteriocins.

Class of Bacteriocin	Subclasses	Molecular Properties	Reference
Class I Lantibiotic	Ia Lanthipeptides Ib Globular and inflexible bacteriocins Ic Sactipeptides	Small, heat-stable bacteriocins (<5 kDa), have a post-translational modification, resulting in the formation of atypical amino acids lanthionine and methyllanthionine.	[33,36,37,38,39]
Class II Non-lantibiotic	IIa Pediocin-like IIb Two peptides IIc Leader less IId Non pediocin-like Single-peptide	Small and flexible bacteriocins (<10 kDa), with an amphiphilic helical structure. These peptides do not contain modified amino acid residues and are pH and heat-resistant.	[13,40,41,42,43,44,45,46]
Class III	IIIa Bacteriolysins IIIb Nonlytic	High molecular weight bacteriocins (>30 kDa), thermolabile and unmodified peptides.	[47,48]

**Table 2:** Bacteriocins produced by lactic acid bacteria inhibit bacteria of interest in veterinary medicine.

Bacteriocin	LAB Producer of Bacteriocin	Susceptible Bacteria	Reference
Enterocin AS-48	*Enterococcus faecalis* UGRA10	*Lactococcus garvieae*	[100]
Enterocin M	*Enterococcus faecium* AL41	*Campylobacter* spp. *Clostridium* spp.	[96]
Enterocin CLE34	*Enterococcus faecium* CLE34	*Salmonella pullorum*	[114]
Enterocin E-760	*Enterococcus durans* *Enterococcus faecium* *Enterococcus hirae*	*Salmonella enterica* serovar Enteritidis *S. enterica* serovar Choleraesuis *S. enterica* serovar Typhimurium *S. enterica* serovar Gallinarum *Escherichia coli* O157:H7 *Yersinia enterocolitica* *Staphylococcus aureus* *Campylobacter jejuni*	[124]
Lacticin 3147	*Lactococcus lactis* DPC3147.	*Streptococcus dysgalactiae,* *Streptococcus agalactiae* *Staphylococcus aureus* *Streptococcus uberis*	[110]
		*Mycobacterium avium* subsp. *paratuberculosis*	[108]
Macedocin ST91KM	*Streptococcus gallolyticus* subsp. *macedonicus* ST91KM	*Streptococcus agalactiae* *Streptococcus dysgalactiae* *Streptococcus uberis* *Staphylococcus aureus*	[125]
Nisin A	*Lactococcus lactic* subsp. *lactis*	*Enterococcus faecalis* ssp. *liquefaciens* *Streptococcus equinus* *Staphylococcus epidermidis* *Staphylococcus aureus* *Streptococcus uberis* *Streptococcus dysgalactiae* *Streptococcus agalactiae*	[77]
		*Streptococcus suis* *Mycobacterium avium subsp. paratuberculosis*	[85] [108]
Nisin A Nisin V	*L. lactis* NZ9700 *L. lactis* NZ9800nisA:M21V	*Listeria monocytogenes*	[87]
Nisin Z	*L. lactis* NIZO22186	*Staphylococcus aureus* *Streptococcus agalactiae*	[78]
Pediocin A	*Pediococcus pentosaceus* FBB61	*Clostridium perfringens*	[119]
